# Dynamic Cerebral Perfusion Electrical Impedance Tomography: A Neuroimaging Technique for Bedside Cerebral Perfusion Monitoring During Mannitol Dehydration

**DOI:** 10.3390/bioengineering12111187

**Published:** 2025-10-31

**Authors:** Weice Wang, Lihua Hou, Canhua Xu, Mingxu Zhu, Yitong Guo, Rong Zhao, Weixun Duan, Yu Wang, Zhenxiao Jin, Xuetao Shi

**Affiliations:** 1 Department of Biomedical Engineering, Shaanxi Provincial Key Laboratory of Bioelectromagnetic Detection and Intelligent Perception, Air Force Medical University, Xi’an 710032, China; 2 Department of Cardiovascular Surgery, Xijing Hospital, Air Force Medical University, Xi’an 710032, China; 3 Institute of Medical Research, Northwestern Polytechnical University, Xi’an 710072, China; 4 Department of Ultrasound Diagnosis, Tangdu Hospital, Air Force Medical University, Xi’an 710038, China

**Keywords:** neuroimaging, brain, cerebral perfusion imaging, bedside monitoring, electrical impedance tomography, total aortic arch replacement

## Abstract

Mannitol dehydration is routinely used to prevent and treat cerebral damage after total aortic arch replacement (TAAR), but existing neuroimaging technologies cannot achieve bedside real-time quantitative assessment of its impact on cerebral perfusion in different patients. This study applied dynamic cerebral perfusion electrical impedance tomography (DCP-EIT), a non-invasive neuroimaging technique, for bedside cerebral perfusion monitoring in TAAR patients during dehydration. Seventeen patients with normal neurological function and nineteen with neurological dysfunction (ND) were enrolled. The variation patterns and differences in perfusion impedance, images, and the relative ratios (RY) of mean perfusion velocity (MV), height of systolic wave (Hs), inflow volume velocity (IV), and angle between the ascending branch and baseline (A_ab_) were analyzed. Results showed DCP-EIT could visualize cerebral perfusion changes, with detected poorly perfused regions showing good consistency with ischemic areas identified by computed tomography (CT). RY of normal patients fluctuated around 0.97–1.04, with no significant difference from baseline. RY of ND patients peaked at 14–20 min after dehydration and remained higher than baseline even at 100 min (*p* < 0.001). DCP-EIT holds potential to optimize individualized cerebral protection strategies for other cerebral damage scenarios and neurocritical care.

## 1. Introduction

Sufficient and stable cerebral perfusion is essential for delivering oxygen and nutrients to brain neurons and timely removing metabolic wastes, thereby ensuring the normal operation of the brain’s various complex functions. Abnormal cerebral perfusion may trigger a series of neurological diseases [1]. Therefore, real-time monitoring of cerebral perfusion is of great significance for the early diagnosis of diseases, the formulation and adjustment of treatment plans, and the assessment of therapeutic efficacy.

Total aortic arch replacement (TAAR) has been identified as a cardiac surgery with a high incidence of cerebral damage [2]. According to the Expert Consensus on Prevention and Treatment of Perioperative Cerebral Injury in Cardiac Surgery, mannitol dehydration is a customary postoperative treatment employed to avert and address cerebral damage-induced poor perfusion [3]. However, further exploration and quantification are required to ascertain the impact of dehydration therapy on cerebral perfusion in patients with normal cerebral function and those with ND. Meanwhile, dynamic monitoring of cerebral perfusion is required to prevent disease deterioration. Consequently, there is an urgent need for technology capable of bedside real-time monitoring of cerebral perfusion in critically ill patients following TAAR.

Current imaging technologies for detecting cerebral perfusion include computed tomography perfusion (CTP), perfusion-weighted magnetic resonance imaging (PWI), and positron emission tomography (PET). Although these technologies can provide high-resolution images, they are limited by bulky equipment, radiation exposure, stringent environmental requirements, and high risks of patient transfer, making bedside real-time monitoring hard [4]. Transcranial Doppler ultrasound (TCD) only reflects hemodynamic information of large intracranial blood vessels and cannot provide images of global cerebral perfusion distribution [5]. Near-infrared spectroscopy (NIRS) enables non-invasive monitoring of regional cerebral oxygen saturation to indirectly evaluate cerebral perfusion. However, its application is restricted by limited inability to detect perfusion changes in deep brain tissues and susceptibility to interference [6]. Consequently, there is an urgent clinical need for a bedside monitoring technology that is convenient, real-time, visualizable, and sensitive to cerebral perfusion changes.

Electrical impedance properties are important physical attributes of biological tissues. They are not only directly related to cell morphology, structure, and arrangement patterns, but also closely associated with functional states such as tissue electrical activity and blood circulation. This enables them to promptly and sensitively reflect the physiological and pathological changes in tissues. Electrical impedance tomography (EIT), an emerging functional imaging technology [7], involves the injection of safe currents into the target area via surface electrodes, the measurement of boundary voltages, and the acquisition of information regarding the spatial distribution of electrical impedance. This information reflects the pathological and physiological states of tissues. EIT has the advantages of non-invasiveness, no radiation, bedside real-time monitoring, and high sensitivity to tissue function alterations. Its potential in real-time observation of physiological and pathological processes has been demonstrated [8]. Holder et al. utilized EIT to monitor impedance changes throughout the seizure process and localize the epileptic focus based on imaging, whilst also performing real-time imaging of neural activity in the brain [9,10,11]. Yang et al. utilized EIT to monitor alterations in impedance in patients with cerebral edema during mannitol dehydration therapy. It was determined that EIT could monitor alterations in water content, which were significantly correlated with intracranial pressure (ICP) [12]. Xu et al. further successfully detected cerebral perfusion abnormalities caused by cerebral infarction using contrast-enhanced EIT. But real-time cerebral perfusion monitoring was not achievable due to the need for contrast agent injection [13]. Cerebral blood flow exhibits pulsatility with the heartbeat, and induces pulsatile changes in cerebral impedance due to the significant difference in resistivity between blood and brain tissue [14]. DCP-EIT is a new functional cerebral perfusion imaging technology developed based on EIT. By accurately acquiring this pulsatile impedance signal and performing image reconstruction, it obtains real-time cerebral perfusion distribution information. It features high speed, high precision, and the capability for long-term monitoring. A DCP-EIT system was developed by our team to rapidly and accurately capture weak signals caused by cerebral blood perfusion [15]. Furthermore, the detection of poor perfusion and the real-time monitoring of perfusion status changes caused by ICP changes were realized [16]. Although our team has verified the technical feasibility of DCP-EIT in animals or healthy volunteers, problems such as how to scientifically and effectively monitor cerebral perfusion in patients in clinical settings and quantitatively evaluate the impact of treatment plans on cerebral perfusion to guide individualized treatment remain unsolved.

This study explores the feasibility and clinical value of DCP-EIT for bedside cerebral perfusion monitoring in patients after TAAR, and conducts the following research: (1) Exploring whether DCP-EIT can achieve visual monitoring of dynamic changes in cerebral perfusion during mannitol dehydration; (2) extracting relevant perfusion parameters to quantify their variation patterns; (3) investigating whether there are differences in the variation patterns of perfusion parameters between patients with normal neurological function and ND; (4) preliminarily exploring whether there is spatial correspondence between perfusion abnormal regions detected by DCP-EIT and brain damage regions indicated by CT. This study provides a foundation for the subsequent wide application of EIT in cerebral perfusion monitoring and the evaluation of treatment efficacy in other patients with cerebral injury, as well as the promotion of individualized cerebral protection strategies and precision medicine.

## 2. Materials and Methods

### 2.1. Study Design and Participants

This was a single-center prospective observational study. Eligible patients in the clinical trial included those who (a) underwent TAAR; (b) were 18–75 years old, regardless of gender; (c) had their hair shaved; (d) received mannitol dehydration therapy; (e) received postoperative DCP-EIT monitoring. In addition, patients were excluded if they (a) had cerebral wounds or acute inflammation, intracerebral or skull metal implants, preoperative examination indicating cerebrovascular malformation or cerebral tumor; (b) were allergic to electrodes; (c) did not sign the informed consent or withdrew midway; (d) had hepatic or renal insufficiency; (e) were deemed unsuitable for inclusion by researchers for other reasons.

**Figure 1:**
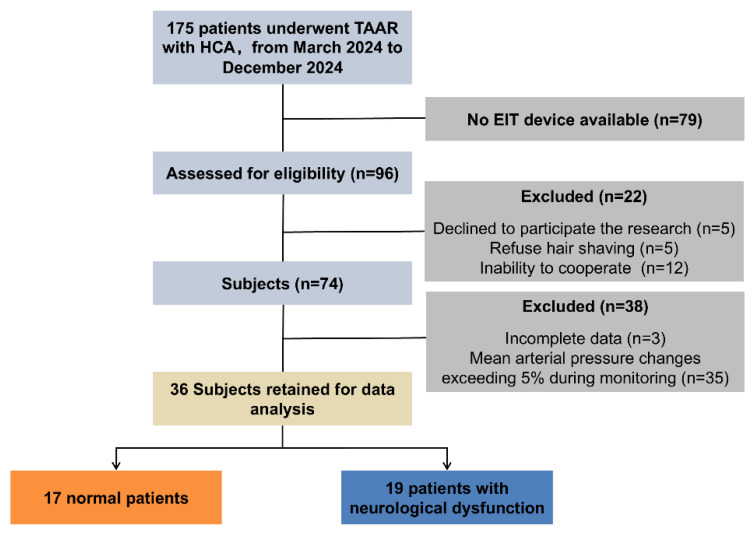
Flowchart of participant inclusion and exclusion.

As demonstrated in Figure 1, a total of 96 patients who underwent TAAR in the Department of Cardiac Surgery of the First Affiliated Hospital of Air Force Medical University and were admitted to the Intensive Care Unit (ICU) from March 2024 to December 2024 were screened in this study, among which 74 patients underwent cerebral perfusion monitoring. Given the impact of significant changes in mean arterial pressure (MAP) on cerebral perfusion, the present study focused on exploring the impact of mannitol dehydration therapy on cerebral perfusion. To this end, 35 cases with MAP relative changes exceeding 5% and 3 cases with incomplete data were excluded. Finally, data from 36 patients were included for analysis.

### 2.2. Standardized Assessment of Neurological Dysfunction and Grouping

For patients admitted to the ICU following TAAR, the Richmond Agitation-Sedation Scale was used to assess sedation levels, the Confusion Assessment Method for Intensive Care Unit was employed to evaluate delirium, the Lovett grading system was used to determine muscle strength, and the Glasgow Coma Scale was used to ascertain whether the patient was comatose, once a day within 7 days after surgery [17,18]. Concurrently, a CT scan was performed as soon as possible when the patient’s condition remained stable to determine whether a new stroke had occurred. All assessments were conducted by two neurology specialists to ensure consistency; in the event of disputes, a third specialist made the final decision. Patients were divided into the normal group and the ND group based on the aforementioned diagnostic results.

### 2.3. Cerebral Perfusion EIT Monitoring Protocol

**Figure 2:**
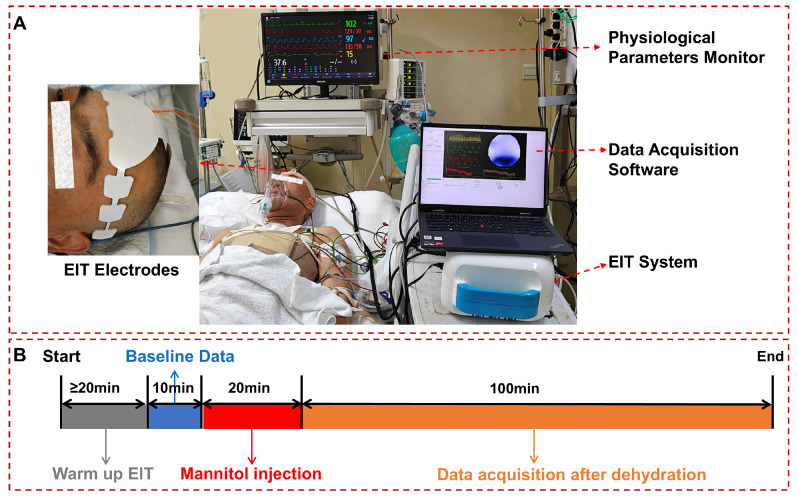
Experimental site diagram and experimental timeline. (**A**): Experimental site of cerebral perfusion monitoring; (**B**): timeline of monitoring and dehydration therapy.

As demonstrated in Figure 2A, the EC-100 PRO, developed by our team, was used for data collection [15]. This system operates within the frequency range of 10–250 kHz, can generate programmable current of 10–1250 μA, and exhibits a signal-to-noise ratio that exceeds 90 dB. In this study, the current was set to 1 mA and the operating frequency was 50 kHz. The system measured 40 frames of data per second to capture cerebral blood perfusion signals during the cardiac cycle.

Cerebral perfusion monitoring of the patient was performed within 24 h postoperatively. Sixteen Ag-AgCl electrodes (EH-PET-16-CS, Yongchuan Technology, Hangzhou, China) were placed at equal intervals on the cross-section 1 cm above the patient’s ears. Meanwhile, the electrodes were wrapped with medical bandages to prevent electrode displacement. Monitoring of cerebral perfusion status during mannitol dehydration therapy was initiated when the patient’s condition was relatively stable.

As shown in Figure 2B, the EIT system was warmed up for at least 20 min before data collection, followed by 10 min of baseline data collection. Then, 0.5 g/kg mannitol was intravenously administered into each patient within 20 min for cerebral dehydration, and data were monitored for 100 min following the completion of dehydration. Concurrently, a physiological parameter monitor was used to assess the patient’s oxygen saturation, heart rate, blood pressure, and respiratory rate.

### 2.4. Dynamic Cerebral Perfusion EIT Imaging

Two validated methods developed by our team were employed to reduce and compensate for artifacts and data loss caused by patient movement during long-term monitoring [19,20]. For DCP-EIT imaging, the raw impedance data were initially subjected to 1–5 Hz band-pass filtering to obtain impedance signals related to cerebral blood perfusion. Subsequently, a finite element model segmented based on human brain CT images and including the actual distribution of electrodes was used for forward problem calculation. Conductivities of the scalp (0.44 S/m), skull (0.012 S/m), and brain parenchyma (0.15 S/m) were utilized as prior information to enhance imaging quality. Finally, the NOSER algorithm was used for inverse problem calculation and reconstruct the conductivity change distribution to obtain cerebral perfusion EIT images [21], as shown in Equation (1):

(1) $$\Delta \sigma ={\left(J^{T}J+\lambda J^{T}J\right)}^{-1}J^{T}\Delta V$$

In this equation, Δ*σ* denotes the change in conductivity distribution between the foreground frame and the background frame, which reflects blood perfusion. Δ*V* signifies the corresponding change in boundary voltage measurements. *J* represents the Jacobian matrix, which encodes the relationship between a unit change in conductivity within each image element and the resulting change in measured voltages. λ is the regularization parameter, crucial for stabilizing the solution of this ill-posed problem by imposing constraints to find a physiologically plausible and smooth image, thereby reducing the impact of measurement noise and model errors. In this study, subsequent cerebral perfusion parameter extraction was conducted by taking the end-diastole of each cardiac cycle as the background frame and the entire cardiac pulsation process as the foreground frame. To enhance the signal-to-noise ratio of EIT and reduce the impact of physiological state fluctuations, 300 frames of data at each time point were used for perfusion status analysis.

The average reconstruction value (ARV) of the region of interest (ROI) in EIT images was calculated using Equation (2):

(2) $$ARV_{ROI}=\frac{\sum_{i=1}^{N}\Delta \sigma_{i}}{N}$$

In this equation, ∆σ_i_ denotes the reconstructed conductivity of the *i*-th unit in the ROI, and *N* represents the total number of units in the ROI. In this study, the brain parenchyma region was selected as the ROI, and its ARV was calculated according to Equation (2).

### 2.5. Extraction of Cerebral Perfusion Parameters

**Figure 3:**
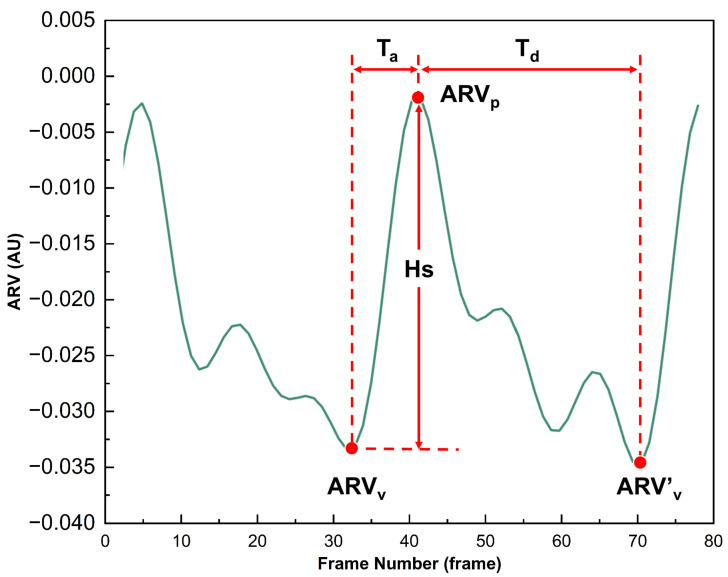
Schematic diagram of cerebral perfusion parameter extraction from a dynamic ARV curve.

As demonstrated in Figure 3, ARV can characterize the dynamic changes in conductivity caused by the congestion state of brain tissue with cardiac pulsation. During the systolic phase of the heart, when blood is pumped into the cranial cavity, the conductivity increases significantly, and the ARV rises from the valley (ARV_v_) to the peak (ARV_p_). This process is defined as the ascending branch, corresponding to a time span of T_a_. Subsequently, during the diastolic phase of the heart, blood gradually flows out of the cranial cavity, which is reflected by the decrease in ARV from the peak (ARV_p_) to the next trough (ARV_v_’). This process corresponds to a time span of T_d_. A complete cerebral perfusion cycle is defined as commencing at ARV_v_ and concluding at ARV_v_’. For this perfusion process, four perfusion parameters (MV, Hs, IV, A_ab_) were extracted. Their extraction equations and corresponding physiological meanings can be found in the Supplementary Materials.

To effectively eliminate differences between individual perfusion parameters and summarize the change patterns of all patients during dehydration, the following data processing method was adopted: the average value X of perfusion parameters during the baseline period was used as the reference value for each patient. Each perfusion parameter Y at various time points during the entire monitoring process was divided by the reference value X, i.e., RY = Y/X, to obtain a series of relative ratios RY (where Y represents MV, Hs, IV, A_ab_, and RY represents RMV, RHs, RIV, RA_ab_). This operation unified the perfusion parameters of different patients at different stages to a relative scale with their respective baseline averages as references. The purpose of this was to significantly improve the comparability of data and facilitate subsequent in-depth analysis and summary of the characteristics of the overall dehydration process.

### 2.6. Statistical Analysis

SPSS 27.0 (SPSS Inc., Chicago, IL, USA) was used for statistical analysis. Categorical variables were expressed as frequency (percentage), and the chi-square test was used to compare differences between different types of patients. For continuous variables, the Shapiro–Wilk normality test was employed to ascertain the conformity of these variables to a normal distribution. In instances where the data conformed to a normal distribution, the data was expressed as the mean ± standard deviation. Conversely, if the data did not conform to a normal distribution, they were expressed as the median (interquartile range). The independent samples *t*-test or independent samples Mann–Whitney test was utilized to compare differences between different types of patients. To evaluate the impact of dehydration on cerebral perfusion, the one-sample *t*-test or one-sample Wilcoxon signed-rank test was used to assess whether there was a significant difference between the relative cerebral perfusion parameters and the baseline data (1) during the monitoring process (*α* = 0.05).

## 3. Results

### 3.1. Demographic and Clinical Characteristics of Patients

A total of 36 patients were included in this study, and they were divided into the normal group (n = 17) and the ND group (n = 19). The comparison results of demographic characteristics and clinical outcome indicators between the two groups are shown in Table 1. For categorical variables, Cramer’s V was used to assess the strength of the association between group status and variables; for continuous variables, Cohen’s d was calculated to measure the standardized mean difference between groups. There were no significant differences in gender, age, or body mass index (BMI) between the two groups (*p* > 0.05). Significant inter-group differences were observed in all neurological dysfunction indicators with the exception of delirium (*p* < 0.05). In terms of clinical outcome indicators, the duration of mechanical ventilation, ICU stay, and treatment cost of patients in the ND group were significantly higher than those in the normal group (*p* < 0.05), while there was no significant difference in total hospitalization time between the two groups (*p* > 0.05). The results indicate that patients with ND exhibit more severe clinical conditions, need prolonged respiratory support time and ICU treatment, and consume more medical resources. This further highlights the importance of real-time monitoring, precise intervention, and efficacy evaluation of postoperative cerebral perfusion status in ND patients.

### 3.2. Changes in Cerebral Perfusion EIT of Normal and ND Patients During Dehydration

**Figure 4:**
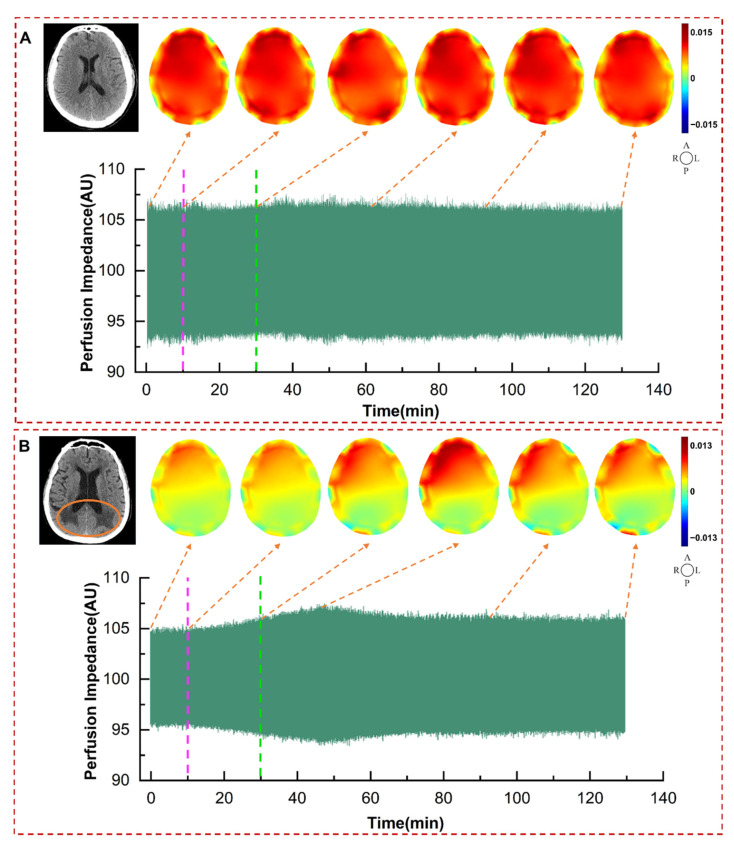
Changes in DCP-EIT of different types of patients during the entire monitoring process. (**A**) Changes in cerebral perfusion impedance, CT image, and cerebral perfusion EIT images of a normal patient at 10 min before dehydration, at the start of dehydration, immediately after dehydration, 30 min post-dehydration, 60 min post-dehydration, and 100 min post-dehydration. (**B**) Changes in cerebral perfusion impedance, CT image, and cerebral perfusion EIT images of a ND patient at 10 min before dehydration, start of dehydration, immediately after dehydration, 16 min post-dehydration, 60 min post-dehydration, and 100 min post-dehydration. (The light purple lines indicate the start of dehydration, and the green lines indicate the end of dehydration).

As shown in Figure 4, cerebral perfusion imaging was performed by taking the end-diastole of the cardiac cycle as the background frame and the end-systole as the foreground frame. Red in cerebral perfusion EIT images indicates an increase in conductivity resulting from blood perfusion, whilst blue denotes a decrease in conductivity. The distribution of red reflects the distribution of cerebral blood perfusion. Figure 4A shows the cerebral perfusion impedance, CT image, and cerebral perfusion EIT images of a normal patient at corresponding time points during the entire monitoring process. There was no regular change in perfusion impedance of the normal patient before and after mannitol dehydration. Furthermore, the cerebral perfusion EIT images at each time point demonstrated that blood was distributed evenly in the entire cerebral region, with no obvious abnormal perfusion areas. And no obvious regular changes were observed in the images during the entire monitoring process.

Figure 4B shows a patient diagnosed by CT with bilateral posterior horn paraventricular white matter ischemia (area outlined by orange circles). The amplitude of perfusion impedance gradually increased with the progression of dehydration, reached the highest value at 16 min after the completion of dehydration. Thereafter, the perfusion signal underwent a gradual attenuation. From the cerebral perfusion EIT images, it was evident that there was a distinct area of poor perfusion in the posterior part of the brain, which roughly corresponded to the ischemic area detected by CT. Meanwhile, with the progression of dehydration, the red color in the EIT perfusion images deepened and the poorly perfused area gradually decreased, indicating that cerebral perfusion was gradually improving with dehydration. The effective cerebral perfusion area and perfusion intensity reached the maximum at 16 min following the completion of dehydration, after which they underwent a gradual decrease. The cerebral perfusion was still improved at 100 min after dehydration compared with the initial state.

**Figure 5:**
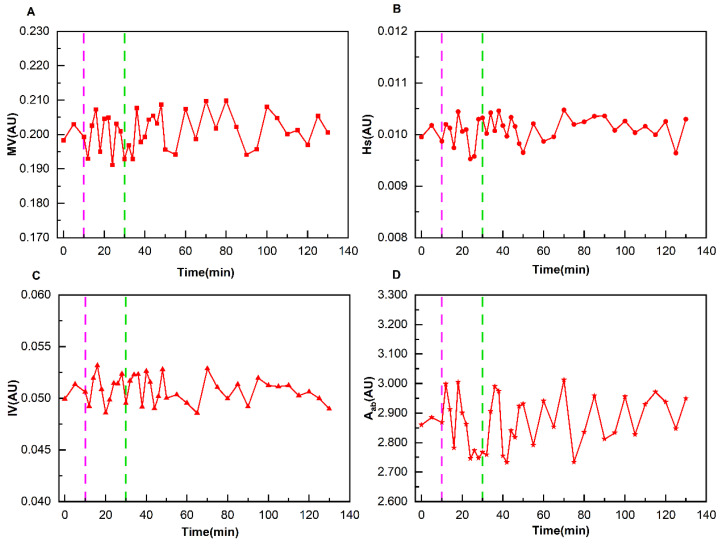
Changes in cerebral perfusion parameters of a normal patient during the entire monitoring process (The light purple lines indicate the start of dehydration, and the green lines indicate the end of dehydration). (**A**) Variation Pattern of MV; (**B**) Variation Pattern of Hs; (**C**) Variation Pattern of IV; (**D**) Variation Pattern of A_ab_.

**Figure 6:**
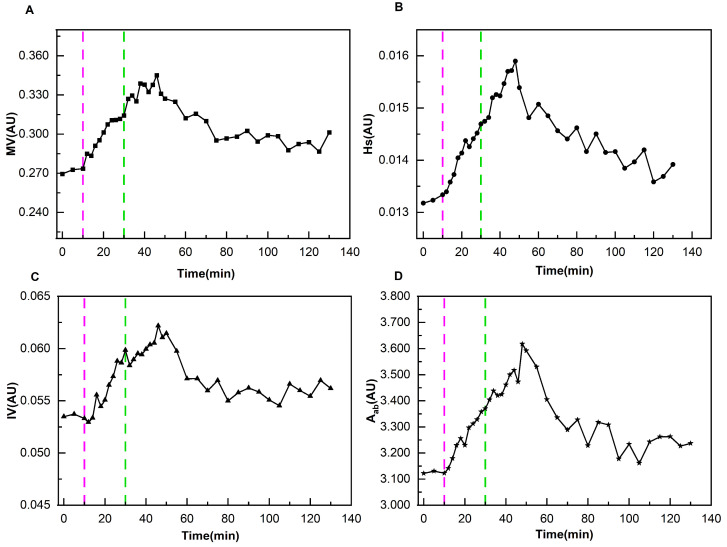
Changes in cerebral perfusion parameters of a ND patient during the entire monitoring process (The light purple lines indicate the start of dehydration, and the green lines indicate the end of dehydration). (**A**) Variation Pattern of MV; (**B**) Variation Pattern of Hs; (**C**) Variation Pattern of IV; (**D**) Variation Pattern of A_ab_.

As demonstrated in Figure 5, it presents the variation patterns of cerebral perfusion parameters of the patient corresponding to Figure 4A during the entire monitoring process. There was no obvious change pattern in the four perfusion parameters during the entire process. The maximum relative change rate of MV, Hs, IV, and A_ab_ was 4.98%. As shown in Figure 6, it presents the change patterns of cerebral perfusion parameters of the patient corresponding to Figure 4B. The relative change rates of MV at immediately after the completion of dehydration, 16 min after the completion of dehydration, 60 min after the completion of dehydration, and 100 min after the completion of dehydration were 15.56%, 26.88%, 11.23%, and 10.79%, respectively. The relative change rates of Hs were 10.89%, 18.62%, 9.46%, and 5.06%, respectively. The relative change rates of IV were 11.85%, 16.20%, 5.09%, and 5.03%, respectively. And the relative change rates of Aab were 7.85%, 11.11%, 5.86%, and 3.59%, respectively.

### 3.3. Changes in Cerebral Perfusion Parameters of the Two Types of Patients During Dehydration

**Figure 7:**
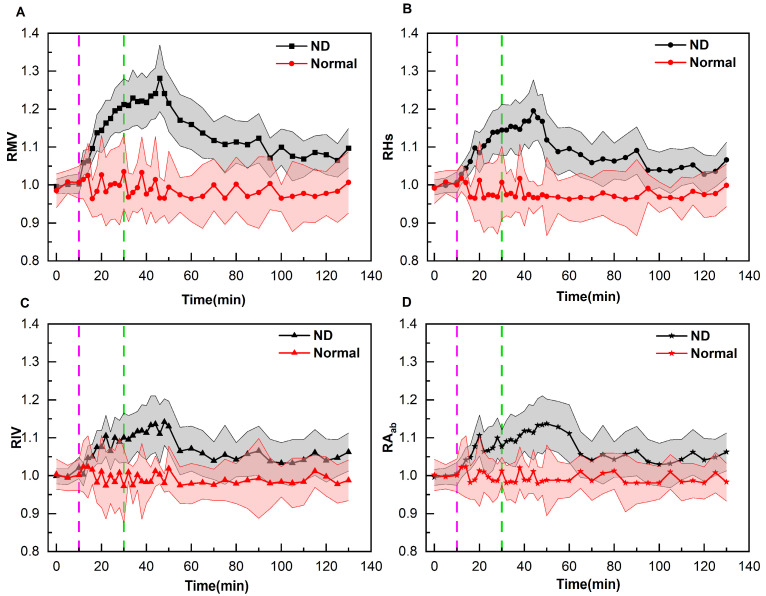
Changes in the mean values of four relative perfusion parameters of normal and ND patients over time (The shaded area indicates the standard deviation. The light purple lines indicate the start of dehydration, and the green lines indicate the end of dehydration). (**A**) Variation Pattern of RMV; (**B**) Variation Pattern of RHs; (**C**) Variation Pattern of RIV; (**D**) Variation Pattern of RA_ab_.

To quantitatively evaluate the therapeutic effect of mannitol dehydration on normal and ND patients, the change patterns of the relative perfusion parameters (RMV, RHs, RIV, RA_ab_) of the two types of patients were investigated, as shown in Figure 7. It can be observed that the RMV, RHs, RIV, and RA_ab_ of normal patients fluctuated around 1. The mean values immediately after the completion of dehydration were 1.04, 1.01, 0.97, and 1.01, respectively, and those at 100 min after the completion of dehydration were 1.01, 1.00, 0.99, and 0.98, respectively. The relative change in RMV exhibited the most significant increase immediately after the completion of dehydration, reaching a maximum value of 1.04. The relative change in RHs was the largest at 10 min following the completion of dehydration, reaching a minimum value of 0.97. The relative change in RIV showed its maximum value immediately after the completion of dehydration, reaching a minimum value of 0.97. The relative change of RA_ab_ demonstrated its maximum value at 16 min after the completion of dehydration, reaching a minimum value of 0.98. The one-sample *t*-test showed that there was no significant difference between RMV, RHs, RIV, RA_ab_ and 1 when their relative changes were the most substantial, with *p*-values of 0.15, 0.07, 0.27, and 0.07, respectively.

The RMV, RHs, RIV, and RA_ab_ of ND patients exhibited obvious change patterns with mannitol dehydration. The parameters remained stable before dehydration and gradually increased after dehydration. The mean values immediately after the completion of dehydration were 1.21, 1.15, 1.10, and 1.08, respectively. As time elapsed, RMV reached a maximum value of 1.28 at 16 min post-dehydration. RHs peaked at 1.20 at 14 min after the completion of dehydration. Additionally, RIV reached a maximum of 1.14 at 18 min post-completion, while RA_ab_ achieved a highest value of 1.14 at 20 min after the completion of dehydration. Thereafter, all perfusion parameters began to gradually decrease. The RMV, RHs, RIV, and RA_ab_ at 100 min after the completion of dehydration were 1.10, 1.07, 1.06, and 1.06, respectively. The one-sample *t*-test demonstrated that there were significant differences between these parameters and 1, with all *p*-values being less than 0.001. The specific values of relative perfusion parameters for the two groups of patients at key time points are presented in Supplementary Material Table S1.

## 4. Discussion

Mannitol dehydration is routinely used in patients after TAAR to prevent and treat potential cerebral damage. Nevertheless, the efficacy of this method in different patients remains uncertain, which hinders the implementation of accurate and effective treatment. Mannitol dehydration is principally employed to reduce cerebral edema, lower ICP, and thereby enhance cerebral perfusion [22,23]. However, there is currently no effective cerebral perfusion monitoring technology suitable for ICU use. This study innovatively used DCP-EIT to realize dynamic real-time monitoring of cerebral perfusion. Concurrently, a perfusion parameter system was established to quantitatively evaluate the therapeutic effect in normal and ND patients. It was found that the detected poorly perfused area by DCP-EIT roughly corresponded with the cerebral infarction area detected by CT. The four relative perfusion parameters of normal patients remained stable during dehydration. The perfusion status of ND patients showed obvious change patterns, with each perfusion parameter reaching its peak at 14–20 min following the completion of dehydration. And the perfusion status was still improved even at 100 min after the completion of dehydration. Through monitoring of patients’ cerebral perfusion, this study quantified the improvement effect of mannitol dehydration in different patients. The findings provide strong support for doctors to formulate and adjust treatment plans and evaluate therapeutic efficacy.

The central aim of this study is to elucidate the impact of mannitol dehydration on the cerebral perfusion of patients after TAAR. It is crucial to exclude patients with significant fluctuations in MAP, as this operation can effectively control confounding variables and ensure the validity of causal inference. The perfusion status of human brain is directly affected by cerebral perfusion pressure (CPP), and CPP = MAP − ICP. Consequently, CPP is also significantly impacted by MAP. Healthy individuals have a well-developed cerebral autoregulation mechanism; when MAP changes within the range of 50–150 mmHg, the human brain can maintain stable cerebral blood flow through vasodilation and contraction [24]. Post-TAAR patients often suffer from cerebral damage due to ischemia-hypoxia caused by the cessation of cardiopulmonary bypass and perfusion abnormalities during the cooling and rewarming stages. This makes the stability of the cerebral circulation extremely vulnerable and leads to impairment of the cerebral autoregulation mechanism. When the cerebral autoregulation mechanism of ND patients is impaired, the capacity to offset the impact of MAP fluctuations may be diminished. In this context, the signals collected by DCP-EIT may be affected not only by dehydration but also by MAP fluctuations. Consequently, patients with MAP changes exceeding 5% were excluded in this study.

Mannitol, a hypertonic solution of macromolecular substances, cannot cross the cell membrane or the intact blood–brain barrier (BBB) [25]. Following intravenous injection of mannitol, plasma osmotic pressure increases significantly. This creates a marked transvascular concentration gradient, which causes small-molecule matter (such as H_2_O, Na^+^, and K^+^) in brain tissue to diffuse into cerebral blood vessels [26]. This diffusion, in turn, reduces brain water content, alleviates cerebral edema, and decreases brain volume, thereby lowering ICP to prevent neurological deterioration [27], and further improving cerebral perfusion. However, improper use of mannitol may cause side effects, including reduced blood volume, electrolyte disorders, increased renal metabolic burden, and even secondary acute rebound of ICP [28]. Therefore, it is crucial to accurately evaluate the efficacy of mannitol dehydration in different patients to optimize the treatment plan and provide a basis for individualized cerebral protection and precision medicine.

By comparing the cerebral perfusion EIT images and CT images of normal and ND patients in Figure 4, it can be seen that the detected poorly perfused area by DCP-EIT roughly corresponds with the cerebral infarction area detected by CT. DCP-EIT overcomes the shortcomings of TCD and NIRS, which cannot reflect global cerebral perfusion or detect poor perfusion in deep brain regions. Another point to note is that due to the critical condition of the patients, postoperative patients could not timely undergo conventional imaging examinations (CT, MRI, PET). In this study, only a subset of patients underwent CT scans when possible, after their condition stabilized postoperatively. Furthermore, conventional imaging techniques are unable to provide real-time, dynamic, and long-term monitoring of changes in perfusion status. The detected alterations in perfusion images by DCP-EIT due to dehydration can help doctors dynamically, intuitively, conveniently, and accurately grasp the improvement of patients’ cerebral perfusion without relevant professional background.

The normal and ND patients exhibited divergent characteristics in cerebral perfusion changes during mannitol dehydration therapy. The four perfusion indicators of the normal group fluctuated around the baseline data, with no significant difference from the baseline data. The presumed mechanism is that mannitol-induced dehydration only mildly reduces ICP. Although this results in alterations to CPP, these are rapidly offset by the intact autoregulatory mechanisms present in the normal group. This ensures that perfusion parameters remain at a fundamentally stable level. In contrast, the perfusion parameters of the ND group exhibited regular changes with dehydration. Each perfusion parameter reached its peak at 14–20 min following the completion of dehydration, and the perfusion status continued to improve even at 100 min after dehydration. The reason for this phenomenon may be that ND patients usually exhibit varying degrees of decompensation of the cerebral autoregulation mechanism. When the ICP of ND patients decreases due to dehydration, the CPP increases.

In neurosurgery, ICP monitoring is usually used to help doctors evaluate the efficacy of dehydration therapy [29,30]. Placing sensors in the ventricle or parenchyma is the gold standard for accurate ICP detection, but it may result in complications such as intracranial hemorrhage and infection [31]. It is important to note that patients following TAAR typically do not undergo invasive ICP monitoring, and not all patients exhibit cerebral damage. Consequently, there is no effective evaluation method for the therapeutic effect of dehydration therapy in both normal and ND patients. By using a high-speed and high-precision EIT system, the sampling frame rate was increased by 40 times compared with that of Yang, Xu et al. [12,13]. This enhancement enabled the successful capture of weak cerebral blood pulsation signals. Concurrently, the limitations of contrast-enhanced EIT, including its invasiveness due to contrast agent injection and inability to realize real-time monitoring, were surmounted.

This study is the first to apply high-speed and high-precision DCP-EIT to bedside monitor the cerebral perfusion of critically ill patients after TAAR. Concurrently, a quantitative evaluation method for mannitol dehydration efficacy was first achieved through the extraction and normalization of four perfusion parameters. On this basis, it was found for the first time that normal and ND patients have different sensitivities to mannitol dehydration, and their change patterns were clarified. These findings provide a tangible basis for clinical practice: for normal patients with stable perfusion, clinicians may avoid unnecessary mannitol dosage increases (reducing risks of electrolyte disorders and renal burden); for ND patients, the 14–20 min post-dehydration peak window can guide timed efficacy assessments, and the sustained perfusion improvement at 100 min supports optimized ICU monitoring schedules (e.g., reducing monitoring frequency).

DCP-EIT provides technical support for the implementation of individualized treatment, precision medicine and the reduction in risks associated with empirical medication, and offers a robust foundation for healthcare professionals to intuitively and scientifically evaluate the efficacy of dehydration, thereby avoiding the wastage of medical resources. The present study will promote the advancement of research in several domains. These include the mechanism of occurrence and development of perioperative cerebral injury, the exploration of cerebral function activities and cerebral science laws, precise management of stroke, and evaluation of therapeutic effects of traumatic brain injury. The study’s findings have the potential to be widely applied in scenarios such as neurocritical care, surgical anesthesia, and emergency rescue to realize bedside non-invasive real-time monitoring of cerebral perfusion in patients.

## 5. Limitations and Future Work

Due to the core imaging principle of inferring changes in internal impedance distribution from boundary voltage variations, EIT still has limitations: the inherent ill-posedness of its inverse problem leads to low spatial resolution. Moreover, it relies on regularization algorithms to balance reconstruction stability and image quality, which may easily introduce image reconstruction errors and make it difficult to capture subtle perfusion abnormalities. To address these limitations, future work will involve developing more advanced image reconstruction algorithms (e.g., integrating CT/MRI prior information), as well as adopting high-density electrode arrays.

To exclude the impact of MAP on cerebral perfusion, patients with MAP changes exceeding 5% were excluded in this study. This relatively strict threshold resulted in 35 patients not being included in subsequent analysis, leading to resource waste. In future studies, the adoption of different MAP change thresholds as inclusion and exclusion criteria will be explored to investigate the differences in cerebral perfusion changes between the two groups. On this basis, the change patterns of DCP-EIT under the combined action of MAP changes and mannitol dehydration will be investigated. Additionally, the perfusion parameters extracted reflect global cerebral conditions. To further refine the research, changes in perfusion parameters between normal brain regions and brain-injured regions in ND patients during dehydration will be investigated, based on lesions identified via CT or MRI.

Meanwhile, the present study is a single-center study. In the future, multi-center studies will be conducted. And cerebral perfusion monitoring in other types of patients in more clinical scenarios will be explored. Subsequent studies have the potential to analyze the prognosis of patients in relation to the varying responses of different patients’ cerebral perfusion to mannitol dehydration. This would facilitate the establishment of a more precise clinical prediction model.

## 6. Conclusions

This study is the first to apply DCP-EIT to bedside monitoring of cerebral perfusion in patients after TAAR during mannitol dehydration. The study found that DCP-EIT can visualize changes in cerebral perfusion. And the detected abnormally perfused areas by DCP-EIT were basically consistent with the ischemic areas indicated by CT. The relative perfusion parameters of normal patients remained relatively stable during dehydration. Conversely, ND patients are sensitive to mannitol dehydration therapy. And the relative perfusion parameters reached their peaks at 14–20 min following the completion of dehydration, and remained significantly higher than the baseline even at 100 min after dehydration. Consequently, DCP-EIT can assist doctors in real-time, visual, and accurate judgment of dehydration efficacy and provide an objective basis for the formulation and adjustment of individualized cerebral protection strategies. This will reduce ineffective interventions, optimize the allocation of medical resources, and avoid potential risks caused by empirical medication. DCP-EIT technology fills the gap in bedside cerebral perfusion monitoring in the ICU and has the potential to be applied to other populations with cerebral damage. This study has the potential to promote the development of critical cerebral protection towards precision, visualization, and individualization. Limitations include the single-center design and sample size restrictions from the 5% MAP threshold exclusion criterion. Future work will focus on multi-center validation and the interactive effects of MAP and dehydration on perfusion parameters to advance clinical application.

## Figures and Tables

**Table 1:** Demographic, neurological function, and clinical outcome characteristics of normal and ND patients after TAAR.

Variables	All (N = 36)	Normal (N = 17)	ND (N = 19)	*p*	Effect Sizes
Sex, male	29 (80.56%)	14 (82.35%)	15 (78.95%)	0.797	0.043
Age, year	51.42 ± 10.83	50.29 ± 11.94	52.42 ± 9.95	0.564	0.195
BMI, kg/m^2^	26.62 ± 3.70	26.05 ± 3.19	27.12 ± 4.12	0.392	0.290
Coma	10 (27.78)	0 (0%)	10 (52.63%)	<0.001	0.587
New stroke	8 (22.22%)	0 (0%)	8 (42.11%)	0.003	0.500
Delirium	2 (5.56%)	0 (0%)	2 (10.53%)	0.169	0.229
Agitation	5 (13.89%)	0 (0%)	5 (26.32%)	0.023	0.380
Abnormal limb movements	8 (22.22%)	0 (0%)	8 (42.11%)	0.002	0.506
Ventilation duration, h	85.50 (118.50)	42.00 (73)	136.00 (176)	0.002	1.237
ICU stay, days	7.50 (6.25)	5.00 (4.00)	9.00 (6.00)	<0.001	1.330
Hospitalization time, days	16.00 (7.00)	16.00 (7.00)	17.00 (9)	0.925	0.321
Cost, RMB	239,064 (115,844)	202,356 (49,588)	286,583 (91,243)	<0.001	1.039

## Data Availability

The data presented in this study are available on request from the corresponding author due to privacy.

## Supplementary Materials

The following supporting information can be downloaded at https://www.mdpi.com/article/10.3390/bioengineering12111187/s1. Material S1: Extraction of Perfusion Parameters. Table S1: Mean Values and Standard Deviations of Relative Perfusion Parameters between the Normal Group and ND Group at Key Time Points.

## Abbreviations

The following abbreviations are used in this manuscript:

TAAR	Total aortic arch replacement
DCP-EIT	Dynamic cerebral perfusion electrical impedance tomography
ND	Neurological dysfunction
MV	Mean perfusion velocity
Hs	Height of systolic wave
IV	Inflow volume velocity
A_ab_	Angle between the ascending branch and baseline
CT	Computed tomography
CTP	Computed tomography perfusion
PWI	Perfusion-weighted magnetic resonance imaging
PET	Positron emission tomography
TCD	Transcranial Doppler ultrasound
NIRS	Near-infrared spectroscopy
EIT	Electrical impedance tomography
ICP	Intracranial pressure
ICU	Intensive Care Unit
MAP	Mean arterial pressure
ARV	Average reconstruction value
ROI	Region of interest
BMI	Body mass index
CPP	Cerebral perfusion pressure
BBB	Blood–brain barrier
