# Supplementary material for: Dynamic Cerebral Perfusion Electrical Impedance Tomography: A Neuroimaging Technique for Bedside Cerebral Perfusion Monitoring During Mannitol Dehydration
Source: Bioengineering (Basel). 2025 Oct 31;12(11):1187. doi: 10.3390/bioengineering12111187 (PMC12649365; doi:10.3390/bioengineering12111187)
Supplement: Supplementary file 1 [file bioengineering-12-01187-s001.zip › bioengineering-3925600-supplementary.pdf]

S1. Extraction of Perfusion Parameters

After calculating the time series signals of ARV from cerebral perfusion EIT images, the following algorithmic process is used to automatically extract feature parameters from each cardiac cycle:

1. Perform automatic valley detection on the continuous ARV signals to identify successive cardiac cycles. Each cycle is defined as the interval from one local minimum of conductivity (end-diastole, ARV_v) to the next local minimum (ARV'_v).
2. Within each cardiac cycle, the system automatically identifies the global maximum point of the cycle (end-systole, ARV_p).
3. Calculate the time intervals T_a and T_d based on the coordinates of the three extracted feature points (ARV_v, ARV'_v, and ARV_p).
4. On this basis, a series of quantitative indicators were extracted [16], including
   - Mean Perfusion Velocity (MV): used to measure the rate of intravascular blood volume change.
   - Height of Systolic Wave (Hs): characterizes the filling degree of cerebral blood vessels and pulsatile blood flow supply.
   - Angle between the Ascending Branch and the Baseline (A_{ab}): reflects the elasticity of cerebral blood vessels. The baseline is defined as a vector starting from ARV_p and parallel to the x-axis.
   - Inflow Volume Velocity (IV): defined as the ratio of Hs to T_a, reflecting the correlation between these two variables.

The calculation formulas for these parameters are as follows:

$$MV = \frac{\sum_{i=1}^{N}(ARV_i - ARV_v)}{T_a + T_d} \quad \text{(S1)}$$

$$H_s = ARV_p - ARV_v \quad \text{(S2)}$$

$$A_{ab} = \arccos\left(\frac{X_v(X_p - X_v)}{\sqrt{X_v^2} * \sqrt{(X_p - X_v)^2 + (Y_p - Y_v)^2}}\right) \quad \text{(S3)}$$

$$IV = \frac{H_s}{T_d} \quad \text{(S4)}$$

where ARV_i represents the average reconstructed value of the ROI in the i-th EIT image; N denotes the total number of EIT images acquired within one perfusion cycle; X_p and Y_p are the x-coordinate and y-coordinate of ARV_p, respectively; and X_v and Y_v are the x-coordinate and y-coordinate of ARV_v, respectively.

Table S1

Table S1. Mean values and standard deviations of relative perfusion parameters between the normal group and ND group at key time points.

Time Points	Groups	RMV	RHs	RIV	RA_{ab}
Baseline	Normal	1.00 ± 0.08	1.00 ± 0.07	1.00 ± 0.08	1.00 ± 0.08
Baseline	ND	1.00 ± 0.07	1.00 ± 0.06	1.00 ± 0.07	1.00 ± 0.07
Immediately after dehydration completion	Normal	1.04 ± 0.10	1.01 ± 0.09	0.97 ± 0.10	1.01 ± 0.04
Immediately after dehydration completion	ND	1.21 ± 0.07	1.15 ± 0.07	1.10 ± 0.06	1.08 ± 0.04
Point of maximum relative change	Normal	1.04 ± 0.10	0.97 ± 0.07	0.97 ± 0.10	0.98 ± 0.04
Point of maximum relative change	ND	1.28 ± 0.09	1.20 ± 0.08	1.14 ± 0.06	1.14 ± 0.07
100 min after dehydration completion	Normal	1.01 ± 0.08	1.00 ± 0.06	0.99 ± 0.05	0.98 ± 0.05
100 min after dehydration completion	ND	1.10 ± 0.05	1.07 ± 0.05	1.06 ± 0.05	1.06 ± 0.05
